# Evaluation of the Antifungal Potential of Grape Cane and Flesh-Coloured Potato Extracts against *Rhizoctonia* sp. in *Solanum tuberosum* Crops

**DOI:** 10.3390/plants12162974

**Published:** 2023-08-17

**Authors:** Francisca Gómez, Catalina Bravo, Isidora Ringler, Christian Santander, Felipe González, Franco Viscarra, Claudia Mardones, Boris Contreras, Pablo Cornejo, Antonieta Ruiz

**Affiliations:** 1 Departamento de Ciencias Químicas y Recursos Naturales, Scientific and Technological Bioresource Nucleus BIOREN-UFRO, Universidad de La Frontera, Temuco 4811230, Chile; 2 Doctorado en Ciencias de Recursos Naturales, Universidad de La Frontera, Temuco 4811230, Chile; 3 Doctorado en Ciencias Mención Biología Celular y Molecular Aplicada, Universidad de La Frontera, Temuco 4811230, Chile; 4 Department of Biochemistry, University of Oxford, South Parks Road, Oxford OX1 3QU, UK; 5 Departamento de Análisis Instrumental, Facultad de Farmacia, Universidad de Concepción, Concepción 4030000, Chile; 6 Novaseed Ltd.a. and Papas Arcoiris Ltd.a., Loteo Pozo de Ripio s/n, Parque Ivian II, Puerto Varas 5550000, Chile; 7 Escuela de Agronomía, Facultad de Ciencias Agronómicas y de los Alimentos, Pontificia Universidad Católica de Valparaíso, Quillota 2260000, Chile

**Keywords:** antioxidant activities, enzymatic activity, biofungicide, photosynthetic traits, plant pathogen

## Abstract

Potato (*Solanum tuberosum*) is one of the most important food crops worldwide, and *Rhizoctonia solani* infection is one of the most common diseases. The objective of this study was to evaluate the antifungal activity of *Vitis vinifera* byproducts (VIDES) and flesh-coloured potato (FCP) extracts against *Rhizoctonia* sp. in potato crops. Photosynthetic traits, phenolic profiles, and antioxidant and enzymatic activities were determined. The VIDES extract showed a 151.4% improvement in stomatal conductance and a 258.5% improvement in the photosynthetic rate compared to the plants without infection. Regarding the enzymatic antioxidant activity, the best response was found in the FCP treatments with 30 min of application, with increases of 25%, 161%, and 450% in ascorbate peroxidase, catalase (CAT), and glutathione reductase (GR) activities, respectively, compared to plants without infection. For the VIDES extract, a 15 min application produced an 83% increase in CAT activity, whereas a 181% increase in GR activity compared to plants without infection was produced after a 30 min application. A similar behaviour was observed for antioxidant compounds, where FCP had a higher concentration of compounds and antioxidant activity. This finding suggests that FCP and VIDES promote the synthesis of plant-defence compounds against *Rhizoctonia* sp. in potato crops, in which the application time is a determining factor.

## 1. Introduction

Potato (*Solanum tuberosum* L.) is one of the most important food crops worldwide [1]. In general, it has a higher source of antioxidant compounds that can reduce the risk of many human diseases [1]. Worldwide, production of this tuber is as high as 380 million tons per year, and a total of 20 million hectares are used in planting, with potato ranking fifth as the main source of energy in the nutrition of the human population [2]. In Chile, approximately fifty thousand hectares are cultivated per year, ranking it as the fourth crop in terms of surface area with the largest number of farmers dedicated to its production, with the region of La Araucanía being the main producer and distributor of potato in the country [3].

Increased consumer demand and crop-associated infections lead to the excessive use of synthetic fungicides and the development of pathogen resistances to the used products, resulting in failures in disease control [4]. While the thermal barrier is one of the main limiting factors for the proliferation of pathogenic fungi, anthropogenic climate change has led to an increase in the ambient temperature, allowing more species of pathogenic fungi to spread and occupy new agroecosystems. This climatological deregulation has detrimental effects in different regions, as some experience increased precipitation and humidity, favouring the growth of fungal species, while others suffer from drought, which also promotes the incidence of other fungal diseases [5]. This situation has also affected potato cultivation, which has transformed into a high-input agricultural production system, requiring a substantial number of agrochemicals for pest management and control. Among the most common diseases is infection by the fungal pathogenic genus *Rhizoctonia* [3,6]. *Rhizoctonia solani* Kühn is a soil-borne plant pathogen that causes significant yield and production losses in potato crops [3]. To prevent or eradicate this infection, fungicides are commonly used. Fungicides are products designed to combat organisms that cause damage to plants; however, the intensive use of fungicides brings adverse consequences for consumers and the environment, causing chemical contamination, reduced soil fertility, and decreased agricultural sustainability by attacking nontarget organisms and producing high resistance in pathogens, disrupting ecosystems and their functioning [3,7,8]. The phenomenon of fungal multiresistance is a major obstacle associated with crop-treatment strategies, which limits the number of effective therapies and drives us to search for and identify new antifungal agents; however, the synthesis of compounds can be very costly and have toxic effects, so the search for natural compounds with antifungal potential is of great interest as a source of study [9].

Alarcón et al. (2021) [10] reported that the use of synthetic fungicides affects the physiology and production of secondary metabolites in potato tubers, such as phenolic compounds, anthocyanins, and hydroxycinnamic acids (HCADs). It has also been demonstrated that fungicides have a direct impact on the primary metabolism of plants, such as photosynthesis and efficient water use [11]. On the other hand, fungicide use also inhibits photosynthesis by destroying chloroplasts, affecting photosystem II activity and chlorophyll biosynthesis, and decreasing plant development [12]. Furthermore, it has been suggested that the use of fungicides is one of the main impediments to the functioning and natural nutrient-uptake capacity of arbuscular mycorrhizal fungi (AMF) in plants [13]. Finally, Zhao et al. (2022) [14] conducted a study describing the negative impact of commercial fungicides on physiological, biochemical, and metabolomic levels in the roots and shoots of wheat plants.

According to the reported background, the generation of new fungicides based on natural products, which guarantee safety in terms of a decrease in adverse effects and a lower environmental impact, has emerged as an alternative of great interest [7]. It is possible to find biological activity in important sources of agronomic wastes, such as in *Vitis vinifera* byproducts. In detail, it has been reported that some grapevine compounds present antifungal activity against *R. solani*; this activity is mainly conferred by their stilbenoids [15,16]. On the other hand, flesh-coloured potato (FCP) has been a major target of study due to its high concentration of anthocyanins and HCADs, compounds with a high antioxidant activity [17,18]. The use of phenolic compounds emerges as an alternative of interest since they possess antifungal activity against *R. solani*, with an in vitro mycelial growth inhibition of up to 67.03% reported [19,20].

Given the characteristics of FCP and *Vitis vinifera* byproducts, the use of these agronomic products as the main components of a potential biofungicide against *Rhizoctonia* sp. is presented as an alternative. Based on these antecedents, it was hypothesised that *Vitis vinifera* and FCP extracts have useful antifungal activity for the control of the disease produced by *Rhizoctonia* sp. in *Solanum tuberosum* crops. The objective of this study was to determine the biofungicidal potential of the byproducts of *Vitis vinifera* and flesh-coloured potato extracts against *Rhizoctonia* sp. in potato crops.

## 2. Results

### 2.1. Variables and Photosynthetic Performance

**Figure 1:**
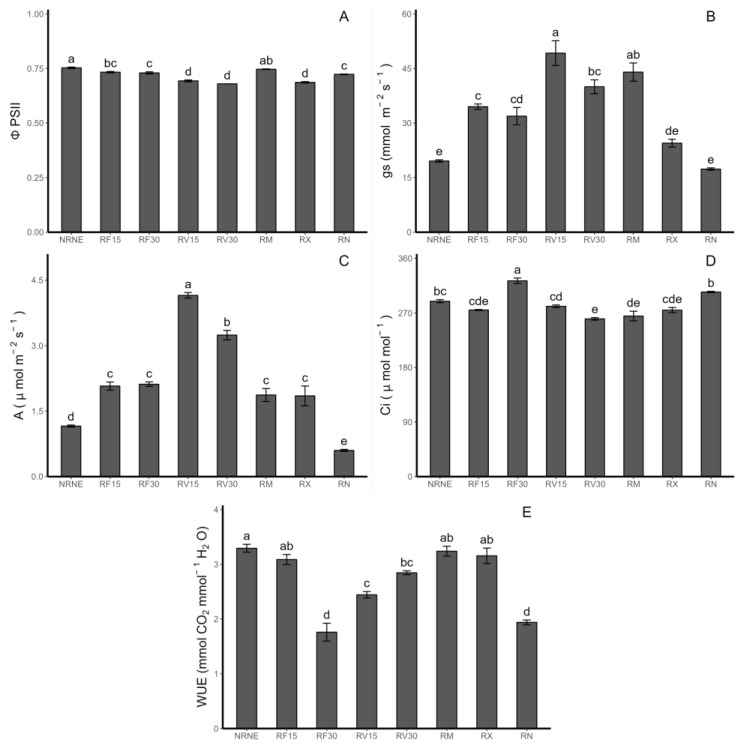
Photosynthetic rate measured in *Solanum tuberosum* leaves of VR808 genotype inoculated or not with *Rhizoctonia* sp. and treated with flesh-coloured potato (FCP) extracts, *Vitis vinifera* (VIDES), or commercial fungicides. The small letters in each value represent the statistically significant difference according to Tukey’s test (*p* < 0.05). In detail, (**A**) photosystem II quantum yield (ΦPSII) (mmol CO_2_ μmol^−1^ photons absorbed); (**B**) stomatal conductance (gs) (mmol H_2_O m^−2^ s^−1^); (**C**) photosynthesis rate (A) (μmol CO_2_ m^−2^ s^−1^); (**D**) CO_2_ leaf internal concentration (Ci) (μmol mol^−1^); (**E**): water use efficiency (WUE) (mmol CO_2_ mol^−1^ H_2_O). Where NR: no *Rhizoctonia* sp. inoculation, NE: no extract or commercial fungicide application, RF: Rhizoctonia and FCP extract, RV: Rhizoctonia and VIDES extract, 15: application for 15 min, 30: application for 30 min, RM: *Rhizoctonia* sp. and MONCUT, RX: *Rhizoctonia* sp. and ReflectXtra, RN: *Rhizoctonia* sp. and extract or commercial fungicide.

Photosynthetic determinations were performed on potato plants. Regarding the performance of photosystem II (ΦPSII), significant differences were observed between the controls, indicating that the ΦPSII efficiency decreased by 4.1% in plants with *Rhizoctonia* sp. compared to the noninoculated plants. On the other hand, a significant difference was also observed between the treatments with the application of the FCP extract and *Vitis vinifera* byproducts (VIDES). Treatment with FCP showed better results compared to the treatments with the VIDES extract application (Figure 1A). For the stomatal conductance (gs: mmol H_2_O m^−2^ s^−1^), significant differences were observed between the treatments with the application of FCP and VIDES. Specifically, VIDES extract application for 15 min increased the stomatal conductance by 42.7% compared to the FCP application for the same amount of time and by 151.4% compared to plants without Rhizoctonia inoculation (Figure 1B). The photosynthetic rate (A) also showed significant differences between the controls, where the control with the presence of *Rhizoctonia* sp. showed a 92.8% lower performance than the treatments with pathogen-free plants. For the treatments with the FCP extract application, no differences were observed between them. In contrast, significant differences were observed among the treatments with the VIDES extract application, with a 28.05% increase in the 15 min application compared to the application of the same extract for 30 min. It is worth noting that RV15 showed significant differences from all treatments, with the highest results for this parameter (Figure 1C). In terms of the internal concentration of CO_2_ (Ci) (Figure 1D), it should be highlighted that the controls did not show significant differences between them, and the application of the FCP extract for 30 min showed significant differences and the highest value among the treatments, with a 19% increase compared to commercial fungicides observed. Finally, in terms of water use efficiency (WUE) (Figure 1E), significant differences were observed among the control groups, with the treatment with Rhizoctonia showing a 69.9% decrease compared to the healthy plant treatments. Furthermore, the application of the FCP extract for 15 min achieved similar results as the commercial fungicides, which did not show significant differences from the healthy plant controls and had the highest results for this parameter among the treatments.

### 2.2. Enzymatic Antioxidant Activity in Potato Leaves

**Figure 2:**
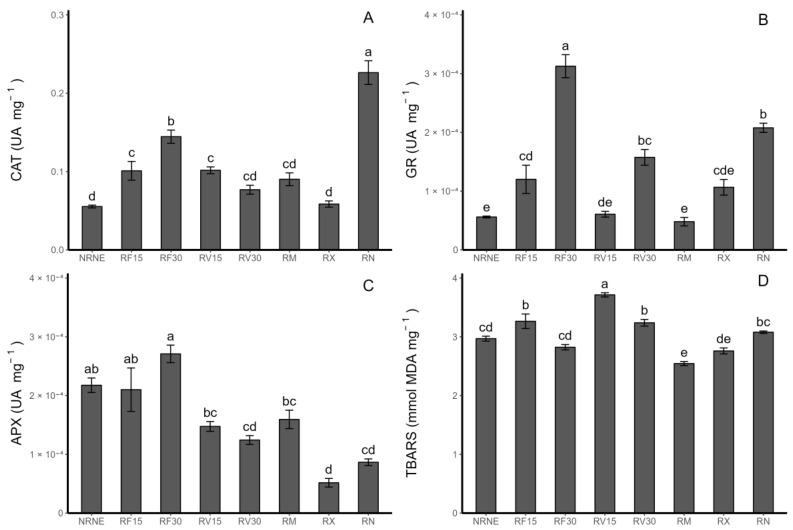
Enzymatic antioxidant activity (UA mg^−1^) in *Solanum tuberosum* leaves of VR808 genotype inoculated or not with *Rhizoctonia* sp. and treated with flesh-coloured potato (FCP) extracts, *Vitis vinifera* (VIDES), or commercial fungicides. Where (**A**) catalase enzyme activity (CAT); (**B**) ascorbate peroxidase enzyme activity (APX); (**C**) glutathione reductase enzyme activity (GR); (**D**) thiobarbituric acid-reactive substance concentration—TBARS (nmol MDA mg^−1^), (MDA) Malondialdehyde formation. NR: no *Rhizoctonia* sp. inoculation, NE: no extract or commercial fungicide application, RF: Rhizoctonia and FCP extract, RV: Rhizoctonia and VIDES extract, 15: application for 15 min, 30: application for 30 min, RM: *Rhizoctonia* sp. and MONCUT, RX: *Rhizoctonia* sp. and ReflectXtra, RN: *Rhizoctonia* sp. and extract or commercial fungicide. Different letters indicate significant differences according to Tukey’s test (*p* < 0.05). Results are given as the mean of the values for each treatment ± the standard error.

Among the treatments, the FCP extract application for 30 min (RF30) showed a higher catalase, glutathione reductase, and ascorbate peroxidase enzymatic activity compared to the treatments with the FCP extract applied for 15 min, VIDES extract applied for 30 and 15 min, and commercial fungicides (Figure 2A–C). Regarding the extracts, the application time of the FCP extract increased the enzymatic activity by 43.1% for catalase (CAT), 160.2% for glutathione reductase (GR), and 28.9% for ascorbate peroxidase (APX) with 30 min of application compared to 15 min of application. On the other hand, in plants treated with the VIDES extracts, the opposite trend was observed for CAT and APX, where the CAT activity decreased by 32.2% and 18.6% in the treatments with a 30 min application time compared to the 15 min application time. In contrast, the VIDES treatment for 30 min produced a 159.3% increase in the GR activity compared to the 15 min application treatment (Figure 2A–C).

With respect to TBARS (Figure 2D), it is worth noting that RF30 had the lowest value of lipid peroxidation compared to the extract application treatments. Additionally, RF30 had the highest enzymatic activity value. The same trend is observed in the case of the Rhizoctonia-inoculated control (RN), which shows a lower lipid peroxidation compared to the control of healthy plants without inoculation. Consistent with this finding, RN exhibited the highest CAT enzymatic activity value among all treatments, and for GR and APX, it was the second highest compared to all the treatments.

### 2.3. Determination of Phenolic Compounds and Their Relationship with the Antioxidant Activity in Potato Leaves

**Figure 3:**
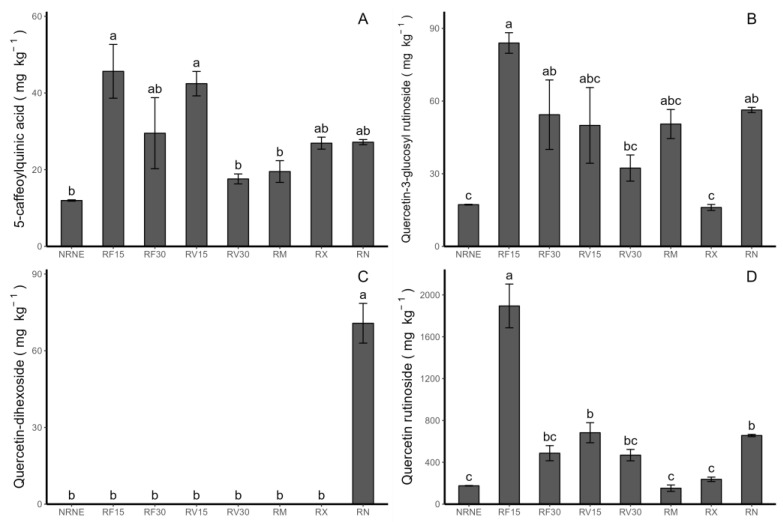
Individual phenolic compounds and HCAD concentration (mg kg^−1^) by HPLC-DAD measured in *Solanum tuberosum* leaves of VR808 genotype inoculated or not with *Rhizoctonia* sp. and treated with flesh-coloured potato (FCP) extracts, *Vitis vinifera* (VIDES), or commercial fungicides. Where (**A**) 5-caffeoylquinic acid; (**B**) quercetin-3-glucosylrutinoside; (**C**) quercetin-dihexoside; (**D**) quercetin-rutinoside. NR: no *Rhizoctonia* sp. inoculation, NE: no extract or commercial fungicide application, RF: Rhizoctonia and FCP extract, RV: Rhizoctonia and VIDES extract, 15: application for 15 min, 30: application for 30 min, RM: *Rhizoctonia* sp. and MONCUT, RX: *Rhizoctonia* sp. and ReflectXtra, RN: *Rhizoctonia* sp. and extract or commercial fungicide. Different letters indicate significant differences according to Tukey’s test (*p* < 0.05). Results are given as the mean of the values for each treatment ± the standard error.

**Figure 4:**
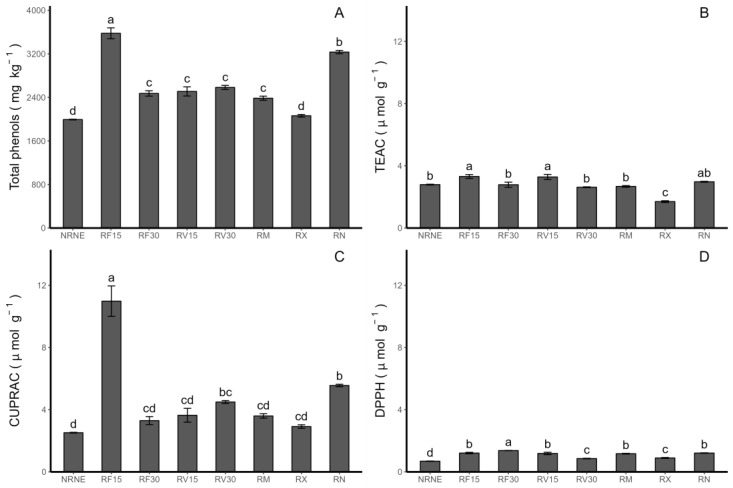
Concentrations of total phenolic compounds and antioxidant activity (AA) in *Solanum tuberosum* leaves of VR808 genotype inoculated or not with *Rhizoctonia* sp. and treated with flesh-coloured potato (FCP) extracts, *Vitis vinifera* (VIDES), or commercial fungicides. Where (**A**) Total phenols determined by Folin–Ciocalteau method; (**B**) AA determined by TEAC method; (**C**) AA determined by CUPRAC (copper-reducing antioxidant capacity) method; (**D**) AA determined by DPPH (2,2-diphenyl-1-picrylhydrazyl) method. NR: no *Rhizoctonia* sp. inoculation, NE: no extract or commercial fungicide application, RF: *Rhizoctonia* and FCP extract, RV: *Rhizoctonia* and VIDES extract, 15: application for 15 min, 30: application for 30 min, RM: *Rhizoctonia* sp. and MONCUT, RX: *Rhizoctonia* sp. and ReflectXtra, RN: *Rhizoctonia* sp. and extract or commercial fungicide. Different letters indicate significant differences according to Tukey’s test (*p* < 0.05). Results are given as the mean of the values for each treatment ± the standard deviation.

Four phenolic compounds were detected, corresponding to one HCAD (5-caffeoylquinic acid) and three flavonols (quercetin-3-glucosylrutinoside, quercetin-dihexoside, and quercetin-rutinoside) (Figure 3A–D; Table 1). In general, the results show higher concentrations of flavonols and lower concentrations of HCAD. Regarding the individual concentrations of 5-caffeoylquinic acid (pick 1), quercetin-3-glucosylrutinoside (pick 2), and quercetin-rutinoside (pick 4), a decrease in concentration was observed, whereas the time of application of the extract increased, which occurred in both extracts, FCP and VIDES. On the other hand, the extract treatments showed the highest values of total phenolic compounds (Figure 4A) in the treatment with RF15.

In general, the RF15 treatment stands out for showing a significant difference compared to the other treatments in terms of flavonols, HCADs, and total phenols, except for quercetin-dihexoside, which is only present in the control treatment in the presence of Rhizoctonia (Figure 3C).

### 2.4. Antioxidant Activities

In terms of the application time of the extracts, in the case of the Trolox equivalent antioxidant capacity (TEAC) method (Figure 4B), the treatments with the FCP and VIDES extracts showed a higher activity with 15 min of application compared to the same extracts applied for 30 min. Specifically, for RF30, the TEAC decreased by 16.08% compared to RF15. For the application of the VIDES extract, the treatment with *Rhizoctonia* sp. inoculum and application of the grapevine extract for 30 min (RV30) decreased by 20% compared to the application of the grapevine extract for 15 min (RV15). Via the copper-reducing antioxidant capacity (CUPRAC) method (Figure 4C), a similar trend was observed, where the application of the FCP extract for 15 min (RF15) had the highest value compared to the rest of the treatments. However, there was also a decrease under the application of the FCP extract for 30 min (RF30) compared to RF15. In terms of the antioxidant activity determined by the 2,2-diphenyl-1-picrylhydrazyl (DPPH) method (Figure 4D), it can be observed that RF30 had a higher activity than RF15, indicating that increasing the application time enhanced the DPPH-determined capacity for FCP. On the other hand, RV30 had a lower activity compared to RV15, indicating the opposite effect, where a shorter application time of the VIDES extract resulted in a higher capacity determined by this method.

### 2.5. Low-Molecular-Weight Organic-Acid Exudation

**Figure 5:**
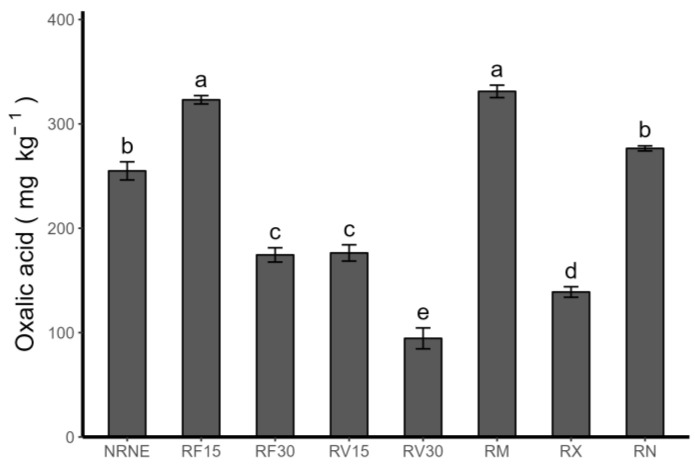
Oxalic acid concentration in rhizosphere of *Solanum tuberosum* crop, VR808 genotype, inoculated or not with *Rhizoctonia* sp., and treated with flesh-coloured potato (FCP) extracts, *Vitis vinifera* (VIDES), or commercial fungicides (mg kg^−1^). Where NR: no *Rhizoctonia* sp. inoculation, NE: no extract or commercial fungicide application, RF: *Rhizoctonia* and FCP extract, RV: *Rhizoctonia* and VIDES extract, 15: application for 15 min, 30: application for 30 min, RM: *Rhizoctonia* sp. and MONCUT, RX: *Rhizoctonia* sp. and ReflectXtra, RN: *Rhizoctonia* sp. and extract or commercial fungicide. Different letters indicate significant differences according to Tukey’s test (*p* < 0.05). Results are given as the mean of the values for each treatment ± the standard error.

Oxalic acid was identified as the only low-molecular-weight acid in the rhizosphere of potato plants. It was identified at concentrations ranging from 94.50 ± 10.03 mg kg^−1^ to 331.15 ± 5.9 mg kg^−1^ (Figure 5). In detail, RV30 presented the lowest concentration value and a significant difference with RV15 and MONCUT, the latter being the treatments that had the highest concentrations among the treatments. It should be noted that the application time appears to be an important factor in the amount of oxalic acid synthesised and exuded by the plant’s roots. Specifically, a decrease of 85.1% was observed in the FCP treatments when the application time increased from 15 to 30 min. The same occurred with the VIDES treatments, where the concentration of oxalic acid decreased by 86.6% when the application time increased from 15 to 30 min.

### 2.6. Agronomic Yield

In terms of the agronomic yield, the mass (g) of the tubers after harvest was determined, with a range of masses between 138 ± 3 g and 185 ± 1 g (Table 2). The control treatment without Rhizoctonia inoculation (NRNE) had the highest mass compared to all the treatments, and it showed a significant difference compared to the control treatment inoculated with Rhizoctonia (RN). Specifically, RN had an 18.1% lower total tuber mass compared to NRNE. On the other hand, no significant differences were observed between the FCP and VIDES extract treatments. It is worth noting that significant differences in the tuber mass were observed between the treatment with the commercial fungicide ReflectXtra (RX) and the treatments with the extracts. RX had an approximately 20% lower tuber mass than RF15, RF30, RV15, and RV30.

### 2.7. Principal Component Analysis

**Figure 6:**
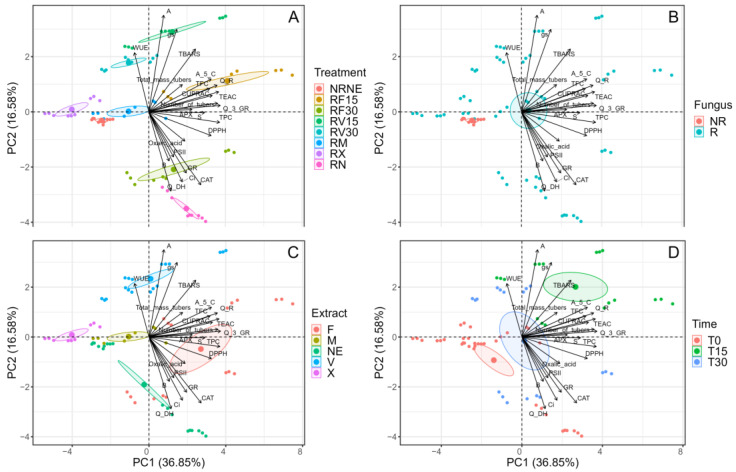
Principal components (PC) for the experimental variables determined in *Solanum tuberosum* of VR808 genotype inoculated or not with *Rhizoctonia* sp. and treated with flesh-coloured potato (FCP) extracts, *Vitis vinifera* (VIDES), or commercial fungicides. The graph shows the distribution of experimental individuals according to PC and grouped according to (**A**) all treatments (**B**) inoculated or not with *Rhizoctonia* sp., where NR: non-inoculated treatments and R: treatments inoculated with the pathogen; (**C**) type of extract or fungicide applied, where NE: no application, F: FCP extract, V: VIDES extract, M: MONCUT fungicide, X: ReflectXtra fungicide; (**D**) time of extract application, where 0 corresponds to treatments without application and to those applied with commercial fungicides.

A principal component analysis was performed (Figure 6), considering the presence of *Rhizoctonia* sp., the application of the extracts (FCP and VIDES), the application of the commercial fungicide (MONCUT (M) or ReflectXtra (X)), and the application time (15 and 30 min, and no application (0)). In relation to the components, it was observed that there is an influence of the antioxidant activity on the treatments with the FCP extract, particularly towards RF15. There is also a correlation between GR, CAT, and oxalic acid in RF30; however, all the antioxidant mechanisms reported are correlated with the use of FCP, and the variation between them is determined by the time of application. On the other hand, an opposite tendency occurs in the use of the VIDES extract, and the influence of the extract treatments on the photosynthetic parameters is observed. This finding suggests that VIDES may be a growth promoter, not an extract that promotes plant-defence mechanisms. Finally, no direct influence of the components on the controls was observed. However, this component analysis shows a difference in correlation between the control plants, which is significant and demonstrates the influence of *Rhizoctonia* sp. on the defence mechanisms and development of potato plants. In relation to the fungus, it was observed that the components only had influence on the treatments in the presence of *Rhizoctonia* sp. In general, it was observed that most of the compounds correlated with the treatments with extract application, suggesting a possible protective action against *Rhizoctonia* sp. as a promoter of plant-defence mechanisms.

## 3. Discussion

In a study conducted by Cayún et al. (2023) [21] on potato plants of the same genotype used in our study (VR808), it was determined that the ΦPSII values for the control treatment of healthy plants were close to 0.75. On the other hand, plants treated with commercial fungicides (ReflectXtra and MONCUT) showed values close to 0.7, where the decrease between the control and the fungicides was not significant. Regarding the quantum yield of the photosystem II values in our study, in the case of the control without the pathogen presence, identical results to the control study reported by Cayún et al. (2023) [21] were observed. The same applies to the results related to the commercial fungicide treatments, with the difference being that in our case, the decrease in the quantum yield of photosystem II between treatments is significant. On the other hand, the treatment in the presence of Rhizoctonia resulted in a significantly decreased quantum yield of photosystem II compared to the healthy control, reaching an average value of 0.73 mmol CO_2_ μmol^−1^ of absorbed photons. This result is similar to what was reported in a previous study for treatments under MONCUT fungicide, indicating that Rhizoctonia negatively impacts the performance of photosystem II, similar to commercial fungicides.

It is worth noting that there is no existing evidence in the literature regarding the results of photosynthetic parameters under similar conditions to those carried out in the present study. Stomatic conductance (gs) is associated with a higher photosynthetic rate (A) because with stomatal opening, more CO_2_ is consumed, decreasing the internal CO_2_ concentration in the leaf (Ci). Many factors may mediate this relationship, such as the use of agriculturally beneficial microorganisms like mycorrhizae, resulting in an improved water use efficiency (WUE) and availability for gas exchange, suggesting an improvement in the stomatal conductance and photosynthetic rate [22,23]. Something very similar occurs in our results regarding the use of natural extracts, such as the VIDES extract, which shows results that demonstrate an increase in stomatal opening and a decrease in the internal CO_2_ concentration, leading to an increase in the photosynthetic rate of plants under this treatment compared to the controls. It is worth noting that these improvements are not only compared to the control with the *Rhizoctonia* sp. presence but also compared to the control of healthy plants without pathogens. This finding suggests that the VIDES extract not only functions as a protector against Rhizoctonia but may also act as a promoter of potato plant development. Finally, Wu et al. (2021) [24] reported the negative influence of *R. solani* on photosynthetic parameters, such as the photosynthetic rate, which is consistent with our results, as the control treatment without application and in the presence of *Rhizoctonia* sp. presented the lowest values of A among the treatments.

Plant enzymes as part of the antioxidant defence system include superoxide dismutase, catalase, glutathione reductase, and ascorbate peroxidase, among others. Together, they form a complex mechanism to reduce and eliminate reactive oxygen species (ROS), generating a different reaction mechanism mediated by the type of stress [25]. During a stress situation, SOD catalyses the elimination of O_2_^−^ into O_2_ and H_2_O_2_, which CAT and APX convert to O_2_ and H_2_O and subsequently eliminate. GR catalyses the reduction of oxidised glutathione to reduced glutathione [26]. The detected trends bring us closer to an explanation of the mechanism of action of the extracts, as in most cases, RF30 surpasses the enzymatic activity of RN. This finding leads us to conclude that antioxidant enzymes act as protectors, preventing the damage to cell membranes caused by Rhizoctonia. In this case, FCP with a 30 min application not only provides protection but also promotes this mechanism, as it shows a higher enzymatic activity and lower lipid peroxidation compared to the Rhizoctonia-inoculated treatment. Youssef et al. (2016) [27] reported that in tomato plants infected with *R. solani* and using AMF as a control agent, infection by *R. solani* led to an increase in the response of antioxidant enzymes due to the elevated ROS levels caused by pathogen infection. They observed a significant decrease in the enzymatic activity in the treatments with biocontrol agents, suggesting a positive regulation of antioxidant enzymes in response to *Rhizoctonia* sp. This finding is consistent with our results regarding enzymatic activity quantification; however, it contradicts the findings from the TBARS analysis. The treatments with a higher enzymatic activity exhibited a lower lipid peroxidation, indicating that the protection system functions preventively or as a defence mechanism. On the other hand, the treatments with a lower enzymatic activity showed a higher lipid peroxidation. Therefore, this positive regulation depends on the mechanism used by the biocontrol agent, which seems to act as an enzymatic promoter for plant defence in the case of the extracts used in our study. It is worth noting that the application time of the extracts appears to be a determining factor in the plant protective action against *Rhizoctonia* sp. In the case of the FCP extract, as the application time increased from 15 to 30 min, the protective effect was enhanced, as we detected a higher enzymatic activity in RF30 than in RF15 and less lipid peroxidation in the RF30 treatments. Based on these results, it is possible to demonstrate that the extracts have a protective effect on plants under oxidative stress caused by the invasion of a pathogen such as *Rhizoctonia* sp., as enzyme synthesis increases during exposure to oxidative stress [26,28].

Our results indicate that the systemic response against *Rhizoctonia* sp. seems to be related to an increase in the synthesis of phenolic compounds in the plant. However, in a study conducted on cucumber plants, an increase in the concentration of 23 phenolic compounds, including HCAD and flavonols, was found in the leaves of plants without systemic symptoms of *R. solani* disease. This finding suggests that the accumulation of these compounds contributes significantly to protection against the pathogen [29], which is consistent with the results reported in the present study, as the treatments with FCP exhibited higher concentrations compared to the controls, suggesting the important role of the extracts as protective agents and providing insights into their mechanism of action. The results are interesting because phenolic compounds such as quercetin-dihexoside are synthesised in plants that are unprotected, exposed to the pathogen, and likely infected by it. It is worth noting that the common factor in the other treatments is the use of biocontrol agents, such as extracts or commercial fungicides, which validates the efficiency of the in vivo protective action of the extracts against the pathogen. Relatedly, it is suggested that the synthesis of quercetin-dihexoside is a specific defence mechanism and/or indicator against Rhizoctonia, which deserves further study to demonstrate its usefulness and function. Studies suggest that the use of commercial synthetic fungicides has a negative impact on tuber secondary metabolites, decreasing the total concentrations of the reported compounds [10]. While the previous results were determined in tubers, they provide evidence of the negative impact of fungicides, which is consistent with our results in leaves, as the same trend occurs. Likewise, according to Nawrocka et al. (2018) [29], in cucumber plants infected with *R. solani* and treated with Trichoderma atroviride (TRS25), the systemic defence response in the plant against pathogen infection was strongly related to the improvement in the synthesis of phenolic compounds, increasing the synthesis of flavonols and hydroxybenzoic acids.

The highest antioxidant activity was detected in the CUPRAC assay. It has been reported that the antioxidant activity responded better to phenolic compounds such as flavonols and HCADs [30]. This finding agrees with other studies where the CUPRAC antioxidant activity increased in plants inoculated with AMF, given its high concentration of flavonols and HCADs [11]. On the other hand, Ruiz et al. (2019) [31] reported that strawberry fruits presented a higher antioxidant activity as observed using the TEAC method, which is explained by the TEAC method showing better antioxidant activity results in the presence of anthocyanins compared to flavonols and HCADs. Finally, in potato leaves of the genotype VR808, Fritz et al. (2022) [11] reported an improvement in the antioxidant activity in plants inoculated with AMF. These symbiotic microorganisms present a protective and promoting role of the antioxidant activity, as do the extracts in the present study. On the other hand, Kang et al. (2015) [32] showed that *R. solani* inhibited Chinese cabbage plant growth, had effects on the photosynthetic parameters, and, in addition, increased the content of the total phenols and antioxidant capacity as determined by the DPPH method. In tomato plants, *R. solani* infection reduced the chlorophyll and anthocyanin contents in the leaves but increased the total phenol and flavanol contents [33]. It is important to note that Fritz et al. (2022) [11] noted a significant decrease in the antioxidant activity in potato plants using ReflectXtra fungicide without the presence of pathogens. This result suggests that the use of fungicides decreases the plant’s self-defence response, and the above reaffirms the idea that these extracts are beneficial for potato plants in the presence of *Rhizoctonia* sp., as they promote the synthesis of antioxidant compounds, increasing their accumulation in the plant and, in turn, the antioxidant capacity [29].

Oxalic acid induces plant resistance to *R. solani* by enhancing defence enzymes and related compounds [34]. Therefore, the exudation of organic acids is not only a solubilisation mechanism but also a defence mechanism against pathogens. Parada et al. (2019) [35] reported higher concentrations of oxalic acid exudates in strawberry plants inoculated with AMF than in our study, suggesting that infection by *Rhizoctonia* sp. influences oxalic acid exudation by decreasing this defence mechanism; however, extracts such as FCP can enhance that mechanism by promoting its exudation.

Regarding the agronomic yield, in the study conducted by Alarcón et al. (2022) [10], the reported biomass of tubers in the VR808 genotype differs from our results in this study. In their case, the control group showed a total biomass of approximately 280 g, while our results show a decrease of 100 g in the biomass of tubers in the control group of healthy plants. This variation may be attributed to the seasonal differences and climatic conditions that the crops faced. It is important to note that the same fertilisation was used in both studies. On the other hand, we identified similarities between our results and those of the treatment with the fungicide MONCUT, indicating that this fungicide had a similar effect in both studies. However, a significant difference was observed in the treatment with the fungicide ReflectXtra, where we reported a lower biomass of tubers compared to the study by Alarcón et al. (2021) [10]. This finding suggests that the presence of the pathogen had a negative impact on our crops, even when commercial fungicides were used. These results support all the previously reported metabolomic results, as it is observed that the pathogen and commercial fungicides have a negative impact on tuber production, whereas the applied extracts protect the plant and yield favourable results similar to those of healthy plants.

Finally, the active compounds of the extracts are related to the physiological and biochemical responses of the plants, and some of the positive effects on plants include increased photosynthetic activity, consumption and efficient use of water, and antioxidant capacity, among others [36]. However, the application time had a similar trend to the difference in extracts; the shorter application time correlated with the antioxidant capacity, and as the time increased, it correlated with the photosynthetic parameters.

## 4. Material and Methods

### 4.1. Reagents

Dimethyl sulfoxide solution 25% *v*/*v*, monopotassium phosphate, urea, thiobarbituric acid (TBA), calcium chloride, and phosphoric acid were obtained from Merck (Darmstadt, Germany). Chlorogenic acid (≥95%), quercetin (≥95%), Trolox (97%), trichloroacetic acid (TCA), and Bradford reagent were acquired from Sigma-Aldrich (Steinheim, Germany). Tween 20 and polyvinylpolypyrrolidone (PVPP) were obtained from Winkler (Santiago, Chile). Finally, oxalic acid was acquired from Supelco (Bellefonte, DE, USA).

### 4.2. Extract Preparation

Flesh-coloured potatoes (CB2011-104 genotype) were provided by Novaseed Ltd.a. (Puerto Varas, Chile). The extraction procedure was carried out according to the description by Ercoli et al. (2021) [18], with modifications. In detail, 500 g of mashed potato was mixed with 1000 mL of extraction (15% glacial acetic acid in absolute ethanol, *v*/*v*). Subsequently, ultrasound was applied for 60 s at 40% amplitude, and the mixture was left in an orbital shaker at 200 rpm for 10 min for homogenisation. Then, it was centrifuged for 10 min at 4 °C and 3000× *g*. The procedure was performed only once and stored at −20 °C in darkness for further treatment. The VIDES extract was made from grape canes of *Vitis vinifera* cv. Pinot noir and was provided by the University of Concepción (Concepción, Chile). Its preparation was carried out according to the description by Escobar et al. (2021) [37], in a 750 L stainless-steel reactor using an ethanol/water solution (80:20 *v*/*v*). Afterwards, it was stored in darkness at 4 °C.

After the preparation of the extracts, it was necessary to select a maintenance solvent without antifungal activity against *Rhizoctonia* sp. based on their properties of solubilising and maintaining the stability of the phenolic compounds in the extracts. In detail, the FCP extract was rotary evaporated and resuspended in a 0.05% *v*/*v* Tween 20 solution. For the VIDES extract, the same procedure was performed, but it was resuspended in dimethyl sulfoxide (DMSO).

### 4.3. Biological Material and Experimental Design

This study was conducted in an outdoor greenhouse with the temperature and light/dark photocycle according to seasonal environmental conditions between December 2021 and February 2022 in the Departamento de Ciencias Químicas y Recursos Naturales of the Universidad de La Frontera, Temuco, Chile, with average temperature highs around 25 °C and average lows around 10 °C. Additionally, the region encountered increased precipitation, with rainy days alternating with sunny periods, and relative humidity ranging between 60% and 80%. A completely randomised design with five replicates per treatment was carried out. The main factors consisted of (i) inoculation of *Rhizoctonia* sp. and a control without inoculation; (ii) application of extracts (VIDES and FCP), commercial fungicides (ReflectXtra (X) or MONCUT (M)), and a control without application; and (iii) time of application of extracts (15 or 30 min) in addition to a control without application (Table 3). The potato genotype used was VR808 (white-fleshed), also provided by Novaseed Ltd.a.

In terms of the factors, the following treatments were created: control without inoculation of *Rhizoctonia* sp. (NRNE), control with inoculation of *Rhizoctonia* sp. (RN), control with inoculation of *Rhizoctonia* sp. and application of the commercial fungicide ReflectXtra (RX), control with inoculation of *Rhizoctonia* sp. and application of the commercial fungicide MONCUT (RM), treatment with *Rhizoctonia* sp. inoculation with 15 min of application of FCP extract (RF15), treatment with *Rhizoctonia* sp. inoculation with 30 min of application of FCP extract (RF30), treatment with *Rhizoctonia* sp. inoculation with 15 min of application of VIDES extract (RV15), and treatment with *Rhizoctonia* sp. inoculation with 30 min of application of VIDES extract (RV30).

The used *Rhizoctonia* sp. strain (accession code: RGM 2705-INIA) was acquired from a national culture collection from Instituto de Investigaciones Agropecuarias (INIA), Chile. For this obtention, a specific isolation for *Rhizoctonia* sp. was performed from a tuber displaying symptoms caused by this pathogen’s disease. On the other hand, the strains were stored through long-term preservation, cryopreservation with glycerol and mineral oil at −80 °C. Before establishing the greenhouse trial, the available *Rhizoctonia* sp. mycelium, obtained from PDA culture with 3-day growth at 28 °C, was inoculated onto 50 potato seeds that were used for planting. Previously, fungal density was determined by the method described by Borie & Rubio (2003) [38]. After inoculation, 10 seeds were immersed in FCP extract, 5 of them for 15 min and the remaining ones for 30 min. In turn, 10 seeds were immersed in grapevine extract, 5 of them for 15 min and the remaining seeds for 30 min. On the other hand, 5 seeds were treated with the commercial fungicide MONCUT, and 5 seeds were treated with the commercial fungicide ReflectXtra, in both cases according to the supplier’s instructions.

For sowing, 11 L pots and peat substrate were used (raw material brown peat Sphagnum H2-H4 von Post * 10% Perlite, pH 5.5). In addition, KH_2_PO_4_ and urea were added as fertiliser [3]. The plants were watered as needed. Day 120 marked the collection of data and photosynthetic parameters using the second-youngest leaf. Harvesting of fresh biomass took place the following day, preserving the same leaves used for data collection by immersion in liquid nitrogen, followed by transfer to a temperature of −80 °C for subsequent analysis. Additionally, the resulting tubers from each treatment were harvested, and their fresh biomass and tuber numbers by pot were determined.

### 4.4. Photosynthetic Determination

The photosynthetic characteristics, such as the internal concentration of CO_2_ (Ci: μmol mol^−1^), photosynthesis rate (A: µmol CO_2_ m^−2^ s^−1^), stomatal conductance (gs: mmol H_2_O m^−2^ s^−1^), and water use efficiency (WUE: mmol CO_2_ mol^−1^ H_2_O), were measured using Targas-1 equipment (PP Systems, Amesbury, MA, USA). In addition, the efficiency of PSII (ΦPSII) was obtained using FluorPen portable equipment (Photon Systems Instruments, Drasov, Czech Republic) using the software FluorPen 1.0. Photosynthetic parameters were measured 2 h after the onset of the photoperiod using the second-youngest leaf.

### 4.5. Enzymatic Antioxidant Activity and Thiobarbituric Acid-Reactive Substances

For enzymatic extracts, 0.3 g samples of leaf tissue were frozen in liquid nitrogen and then ground in 0.9 mL of solution containing 0.1 mol L^–1^ phosphate buffer (pH 7.0) and 2.5% (*w*/*v*) polyvinylpyrrolidone [21]. The supernatant obtained was used for protein quantification via the Bradford method [39]. The enzymatic determination of catalase, ascorbate peroxidase, and glutathione reductase was performed according to the description by Aguilera et al. (2020) [40]. A microplate spectrophotometer was used for all determinations (Synergy HTX UV-visible Biotek, Winooski, VT, USA).

Lipid peroxidation was determined by malondialdehyde (MDA) formation using the thiobarbituric acid method according to the description by Du & Bramlage (1992) [41] with modifications. After extraction, 300 µL of supernatant was taken, and 1.2 mL of a solution containing 20% TCA and 0.5% TBA was added. The mixture was incubated at 95 °C on a hot plate for 30 min, after which time it was removed and cooled in an ice bath and then centrifuged at 10,000× *g* for 10 min at 4 °C. Finally, absorbance readings were taken at 440, 532, and 600 nm in a Synergy HTX UV-visible microplate spectrophotometer Biotek (Winooski, VT, USA).

### 4.6. Identification and Quantification of Phenolic Compounds in Leaves

The extraction and identification of phenolic compounds was carried out according to the description by Fritz et al. (2022) [11]. High-performance liquid chromatography with diode array detection (HPLC-DAD) equipment Shimadzu (Tokyo, Japan) equipped with a quaternary pump (LC-20AT), a degassing unit (DGU-20A5R), a column oven (CTO-20A), an autosampler (SIL-20A), and a UV-Vis diode array detector (SPD-M20A) was used for identification and quantification using a Kromasil C_18_ column (100 × 4.6 mm, 2.5 µm) with a Nova-Pak C_18_ precolumn (22 × 3.9 mm, 4 µm; Waters) at 40 °C. HCADs were quantified at 320 nm and flavonols at 360 nm using chlorogenic acid and quercetin as external calibration standards, respectively. Identity assignments were performed according to the description by Nova et al. [42] using an HPLC-DAD-QTOF-MS/MS Compact (Bruker Daltonics GmbH, Bremen, Germany). Instrument control and data collection were carried out using Compass DataAnalysis 4.4 SR1 (Bruker Daltonics GmbH).

### 4.7. Determination of Total Phenolics and Antioxidant Activity

The determination of total phenols, Trolox equivalent antioxidant capacity (TEAC), copper-reducing antioxidant capacity (CUPRAC), and antioxidant activity by the 2,2-diphenyl-1-picrylhydrazyl (DPPH) method was performed as described by Parada et al. (2019) [35] in the extracts used for phenolic compound determination, using chlorogenic acid as a standard for the quantification of total phenols. On the other hand, the Trolox standard was used for the TEAC, CUPRAC, and DPPH methods. Measurements were performed on a Synergy HTX UV-visible microplate spectrophotometer (BioTek, Winooski, VT, USA) at 750 nm for the determination of total phenols, 734 nm for TEAC, 450 nm for CUPRAC, and 517 nm for DPPH.

### 4.8. Exudation of Low-Molecular-Weight Organic Acids

Low-molecular-weight organic-acid (LMWOA) concentrations were determined according to the method described by Parada et al. (2019) [35] with modifications. Rhizosphere samples (0.5 g) were dissolved in 10 mL of 0.2 M calcium chloride, then centrifuged at 4000× *g* for 15 min at 4 °C, and the supernatant was retained. Chromatographic analysis was carried out using HPLC-DAD with characteristics described by Parada et al. (2019) [35]. In detail, a Kromasil C_8_ column (250 × 4.6 mm, 5 µm) and a Kromasil C_8_ precolumn (100 × 4.6 mm, 5 µm) were used with 0.2 N phosphoric acid, pH 2.1, as the mobile phase at a flow rate of 1.0 mL min^−1^ in an isocratic system at 30 °C. Quantification was performed at 210 nm by external calibration with oxalic acid as a standard.

### 4.9. Statistical Analysis

All statistical analyses were performed and figures were prepared in R version 4.2.1. One-way ANOVA was used to test for significant differences between measurements of each experimental variable. For the variables with significant differences, the means were compared using the Tukey HSD multiple range test with the package “agricolae” v. 1.3.5. Moreover, the dataset was subjected to principal component analysis (PCA). Confidence ellipses (group means) by differences in treatments, fungus, extract, and time were also generated using the packages “FactoMineR” v. 2.7 and “factoextra” v. 1.0.7.

## 5. Conclusions

In detail, the FCP extract increases the concentration of compounds related to the antioxidant defence in the leaves of potato plants for the control of *Rhizoctonia* sp. On the other hand, the VIDES extract primarily enhances the photosynthetic parameters of potato plants in those artificially infected with *Rhizoctonia* sp. The above suggests that the extracts exhibit differentiated activities. FCP appears to be associated with the synthesis of plant-defence compounds, whereas VIDES enhances the processes of the plant’s primary metabolism, potentially leading to an increase in the plant’s growth rate as it improves photosynthetic processes.

The results highlight the positive protective effects of the extracts against the pathogen, which are validated through their agronomic performance. However, it is important to investigate the mechanism employed by the extracts for crop protection, as well as to optimise the type and timing of application. Such work will lead us to the development of applied science and an efficient, useful, and marketable product. It seems that the action of the extracts is mediated by these variables, as well as by the plant–extract relationship that can be established depending on the applications.

## Figures and Tables

**Table 1:** Identification of phenolic compounds in *Solanum tuberosum* leaves of VR808 genotype inoculated or not with *Rhizoctonia* sp. And treated with flesh-coloured potato (FCP) extracts, *Vitis vinifera* (VIDES), or commercial fungicides by HPLC-DAD-ESI-MS/MS.

Peak	Compound	٨max (nm)	[M−H]^−^	Productions
1	5-caffeoylquinic acid	325	353.1	191.1
2	quercetin-3-glucosylrutinoside	352	771.7	301.0
3	quercetin-dihexoside	352	625.0	301.0
4	quercetin-rutinoside	352	609.1	301.0

**Table 2:** Agronomic yield of *Solanum tuberosum* tubers of genotype VR808 inoculated or not with *Rhizoctonia* sp. and treated with extracts from flesh-coloured potato (FCP), *Vitis vinifera* (VIDES), or commercial fungicides. Where NR: no *Rhizoctonia* sp. inoculation, NE: no extract or commercial fungicide application, RF: Rhizoctonia and FCP extract, RV: Rhizoctonia and VIDES extract, 15: application for 15 min, 30: application for 30 min, RM: *Rhizoctonia* sp. and MONCUT, RX: *Rhizoctonia* sp. and ReflectXtra, RN: *Rhizoctonia* sp. and extract or commercial fungicide. Different letters indicate significant differences according to Tukey’s test (*p* < 0.05). Results are given as the mean of the values for each treatment ± the standard error.

Name	Tubers (g)
NRNE	185 ± 1 a
RF15	171 ± 1 c
RF30	167 ± 2 bc
RV15	167 ± 4 bc
RV30	172 ± 3 c
RM	160 ± 1 b
RX	138 ± 3 d
RN	157 ± 2 b

**Table 3:** Experimental design treatments in *Solanum tuberosum* leaves of VR808 genotype inoculated or not with *Rhizoctonia* sp. and treated with flesh-coloured potato (FCP) extracts, *Vitis vinifera* (VIDES), or commercial fungicides.

Name	Inoculation	Type of Compound Used	Time of Application	Number of Replicates
NRNE	without *Rhizoctonia* sp.	Without compound	Does not apply	10
RF15	with *Rhizoctonia* sp.	Application of FCP	15 min	5
RF30	with *Rhizoctonia* sp.	Application of FCP	30 min	5
RV15	with *Rhizoctonia* sp.	Application of VIDES	15 min	5
RV30	with *Rhizoctonia* sp.	Application of VIDES	30 min	5
RM	with *Rhizoctonia* sp.	Application of MONCUT	Application by supplier	5
RX	with *Rhizoctonia* sp.	Application of ReflectXtra	Application by supplier	5
RN	with *Rhizoctonia* sp.	Without compound	Does not apply	9

## Data Availability

The data presented in this study are available on request from the corresponding author.
